# DIO3 associates with sorting nexins and endosomal trafficking networks in ovarian cancer

**DOI:** 10.1007/s13402-026-01236-z

**Published:** 2026-06-02

**Authors:** Daniel Maman-Shinar, Shachar Vanmak, David Bern, Nir Ben-Tal, Amit Rosemarin, Dotan Moskovich, Noi Haim, Ulrich Schweizer, Marietta Fabiano, Avivit Weisz, Aviva Katzav, Debora Kidron, Mario Beiner, Aula Asali, Yael Hants, Maya Gottfried, Osnat Ashur-Fabian

**Affiliations:** 1 Translational Oncology Research, The Oncology Institute, Meir Medical Center, Kfar-Saba, 44821 Israel; 2 Department of Human Genetics and Computational Sciences, Gray School of Medical Sciences, Gray Faculty of Medical & Health Sciences, Tel Aviv University, Tel Aviv, Israel; 3 School of Neurobiology, Biochemistry and Biophysics, George S. Wise, Faculty of Life Sciences, Tel-Aviv University, Tel-Aviv, Israel; 4 Institut für Biochemie und Molekularbiologie, Universitätsklinikum Bonn, Rheinische Friedrich-Wilhelms-Universität Bonn, Bonn, Germany; 5 The Pathology Institute, Meir Medical Center, Kfar-Saba, Israel; 6 Gray School of Medicine, Gray Faculty of Medical & Health Sciences, Tel Aviv University, Tel Aviv, Israel; 7 Division of Gynecologic Oncology, Meir Medical Center, Kfar Saba, Israel; 8 The Oncology Institute, Meir Medical Center, Kfar Saba, Israel

**Keywords:** Deiodinases, Ovarian cancer, Recycling, Trafficking

## Abstract

**Puropose:**

Type 3 iodothyronine deiodinase (DIO3) is a membrane-associated enzyme that inactivates thyroid hormones and promotes high-grade serous ovarian cancer (HGSOC) progression. Although DIO3 undergoes continuous recycling between the plasma membrane and endosomal compartments, the mechanisms governing its intracellular routing and functional stability remain unknown.

**Methods:**

We used Human ovarian cancer cells (ES-2, OVCAR3, OVSAHO, KURAMOCHI, NCI/ADR (NAR)), ovarian cancer tumors and normal tissues from patients, immortalized fallopian tube cells (FT109, FT237 and FT282) and HEK293T cells. DIO3 was knocked-down (shRNA) or overexpressed (CRISPR activation system). Flow cytometry (cell counts and apoptosis assay), proteomics, co-immunoprecipitation, proximity ligation assay, confocal imaging in the presence of endosomal transport inhibitors, and AlphaFold-based structural modeling were performed.

**Results:**

We identify sorting nexins SNX2 and SNX6 as components of the DIO3 trafficking machinery in ovarian cancer. Computer-based structural analysis provide a potential interface for SNXs association, requiring intact linker and catalytic domains of DIO3. Studies in ovarian cancer cells suggest that SNX6 is linked with DIO3 progression through endosomal and Golgi compartments, whereas SNX2 is associated with its recycling to the plasma membrane via RAB11-positive vesicles. Disruption of these pathways destabilizes DIO3-SNXs colocalization and selectively impairs proliferation and survival of DIO3-overexpressing cells.

**Conclusion:**

These findings establish intracellular trafficking as a fundamental determinant of DIO3 stability and function and uncover sorting nexin-associated routing as a previously unrecognized dependency and therapeutic vulnerability in ovarian cancer cells.

**Supplementary Information:**

The online version contains supplementary material available at 10.1007/s13402-026-01236-z.

## Background

Deiodinase type 3 (DIO3) functions as a critical enzyme for inactivating triiodothyronine (T3) and thyroxine (T4), thereby modulating local thyroid hormone signaling [1, 2]. This homodimeric enzyme has been linked to tumor growth in several aggressive malignancies [3, 4]. We recently established a similar pro-mitogenic role for DIO3 [5, 6] in high grade serous ovarian cancer (HGSOC), the most common aggressive subtype of ovarian cancer, which frequently originates from the fallopian tube epithelium [7, 8]. DIO3 continuously recycles between the plasma membrane and endosomal compartments, enabling precise spatiotemporal control of endogenous thyroid hormone metabolism [9–11]. However, the exact molecular machinery governing this trafficking remains unexplored. Given DIO3’s role in tumors, defining the mechanisms that regulate its intracellular transport is essential for understanding its broader biological significance.

Sorting nexins (SNXs) play a central role in endosome recycling of numerous proteins to and from the cell membrane, endosomal sorting and regulation of degradation [12, 13]. These proteins contain a highly conserved lipid-binding phox homology (PX) domain, allowing SNXs to bind to specific phosphoinositides which mark different membrane surfaces. Several SNX proteins, including SNX2, SNX4, SNX5 and SNX6, contain an additional Bin/Amphiphysin/Rvs (BAR) domain, a dimerization motif. These BAR regions that enhance membrane binding, membrane remodeling, and protein-protein interactions, include the ability to form heterodimeric complexes between SNX-BAR pairs [14–16]. These complexes were reported to be composed of heterodimeric combinations of SNX2 with SNX5 or SNX6 [17] and to play a critical role, not only in endosome–to–plasma membrane recycling, but also in endosome to the trans-Golgi network (TGN), known as retromer transport [18–20].

DIO3 recycling through the endosomal system, together with the fact that both DIO3 [21] and SNXs require phospholipids for their activity, led us to speculate that these proteins may interact. In this study, we identified that SNX2 and SNX6, which are regulated by cancer-related signaling cascades [22], associate with DIO3. We further elucidated their colocalization with DIO3 movement through early and late endosomes, recycling compartments, and the Golgi apparatus in ovarian cancer. These findings may shed light on a previously unrecognized layer of DIO3 regulation and offer a framework for further exploration of thyroid hormone signaling control at the subcellular level.

## Methods

### Reagents and antibodies

Primary and secondary antibodies are detailed in Supplementary Table S1. EGA, an inhibitor of early to late endosome trafficking, Hygromycin, Blasticidin and Puromycin and Aurothioglucose (ATG), a non-specific DIO3 inhibitor (Merck, Darmstadt, Germany). 2-(4-Fluorobenzoylamino)-benzoic acid methyl ester (EXO1), a reversible inhibitor of endoplasmic reticulum (ER)-Golgi trafficking (R&D Systems, Minneapolis, MN, USA). Brefeldin A (BFA), a Golgi-to-ER transport inhibitor (Tocris, Abingdon, United Kingdom). Primaquine phosphate (PMQ), inhibits recycling-endosome function and prevents return of membrane proteins to the cell surface (Cayman chemical, Ann Arbor, Michigan, USA). Monodansylcadaverine (MDC), an inhibitor of clathrin-mediated endocytosis (Santa Cruz, Dallas, Texas, USA). Golgi apparatus staining (ab139483, Abcam, Cambridge, MA, USA). ITYR-DBRMD, synthesized as previously described [6], was used as a specific DIO3 inhibitor. All inhibitors were dissolved in DMSO. Working concentrations and incubation times were selected following optimization experiments.

### Cell lines

ES-2 (CRL-1978) and OVCAR3 (HTB-161) cells were purchased from the ATCC. OVSAHO, KURAMOCHI and immortalized fallopian tube cells (FT109, FT237 and FT282) were provided by Dr. Ruth Perets (Rambam Medical Center, Haifa, Israel). NCI/ADR (NAR) cells were provided by Prof. Dan Peer (Tel Aviv University, Tel Aviv, Israel). HEK293T cells were provided by Dr. Yaara Oren (Tel-Aviv University, Tel-Aviv, Israel). HGSOC cells were cultured in complete RPMI medium with 10% FCS. 1% MEM was added for KURAMOCHI and 0.01 mg/ml Insulin and 20% FCS was added for OVCAR3 cells. Fallopian tube cells were grown in DMEM F-12 supplemented with 1% MEM. HEK293T cells were grown in high-glucose DMEM. COS7 cells, stably transfected with mouse DIO3-Flag cDNA cloned into pcDNA3 vectors [23], were cultured in DMEM with 10% FCS. Cell authentication was verified by STR/mutation profiling, and mycoplasma testing was performed periodically.

### DIO3 knockdown (DIO3-KD)

ES-2 cells were seeded (0.5 × 10^6^/6 well plates) and stably transfected with a pool of 3 human DIO3 shRNA plasmids (Cat. # sc-77150-SH) or a nonspecific scrambled control shRNA plasmid (Cat. # sc-108060, Santa Cruz). Transfections were performed following manufacturer’s instructions with shRNA transfection reagent (Cat. # sc108061, Santa Cruz). After selection with puromycin and clones’ isolation, successful stable inhibition was confirmed at the RNA (Real-time PCR) and protein levels (Western blots) using DIO3-validated antibody, as we previously described [5].

### DIO3 CRISPR activation

HEK293T cells were seeded (2 × 10^5^/6 well plates) and stably transfected with a synergistic activation mediator (SAM) CRISPR transcription activation system, which provides a robust system to maximize the activation of endogenous gene expression [24]. CRISPR activation plasmids which enable the identification and upregulation of DIO3 by utilizing a D10A and N863A deactivated Cas9 (dCas9) nuclease fused to a VP64 activation domain, in conjunction with sgRNA (MS2), a DIO3-specific sgRNA engineered to bind the MS2-P65-HSF1 fusion protein (Cat. # sc- 403729-ACT, Santa Cruz) or a control CRISPR activation plasmid (Cat. #sc-437275, Santa Cruz). Transfections were performed following manufacturer’s instructions with Transfection Reagent – UltraCruz^®^ (Cat. # sc-395739, Santa Cruz). After selection with Hygromycin, Blasticidin and Puromycin clones were isolated and successful stable inhibition was confirmed at the protein levels (Western blots).

### Flow cytometry

Absolute cell counts and Annexin V/Propidium Iodide (Annexin V/PI) were performed using MACSQuant flow cytometer (Miltenyi Biotec, Bergisch Gladbach, Germany), as detailed before [25].

### Human tissue samples

Tumor tissues were collected from HGSOC patients at diagnosis undergoing primary debulking surgery as well as from normal fallopian tube (FT) fimbria tissues from non-oncological patients undergoing laparotomy or ultrasound guided paracentesis treated at the Obstetrics and Gynecology Department, Meir Medical Center, Israel. HGSOC diagnosis was confirmed based on clinical and pathological characteristics. The tissues were formalin fixed paraffin embedded (FFPE) and 4 μm sections were cut from the tissue blocks at the pathology department, Meir Medical Center. Tissues were collected upon informed consent, in compliance with Meir Medical Center Institutional Review Board (IRB) approval, in accordance with the Declaration of Helsinki (Study 0305-16-MMC).

### Western blot (WB)

Total proteins were collected, separated and analyzed as detailed before [25]. β actin was used as loading control. Bands were measured by LAS3000 imaging system and analyzed by Multigauge v3.0 software (Fujifilm Life Science, Tokyo, Japan).

### Co-immunoprecipitation (Co-IP)

Total proteins or protein fractions (cytosol, membrane and nucleus), using Fraction PREP TM Cell Fractionation kit (BioVision, Milpitas, CA, USA) as described in [26], were extracted from HGSOC cells (~ 4 or 8 × 10^6^ cells each line, respectively). Proteins were concentrated using Amicon Ultra 3 kDa centrifugal filters (Merck, Darmstadt, Germany). Lysates were mixed with 5X IP low buffer (Active Motif, Waterloo, Belgium) diluted with 1X DDW supplemented with protease inhibitor (Active Motif) and incubated overnight at 4 °C with the primary antibody under gentle rotation, then rotated 1 h on ice and 5 min at room temperature. Protein G magnetic microbeads (Miltenyi Biotec, Bergisch Gladbach, Germany) were added at 2 µg antibody:50 µl beads and rotated 1 h on ice. Immunoprecipitation was done using µMACS columns (Miltenyi Biotec). The lysates were loaded to the columns three times, washed eight times with 1X IP low buffer, then rinsed with 20 mM Tris-HCL pH 7.5 with 20 µl of 1X sample buffer preheated to 85 °C. Elution was collected using 1X sample buffer preheated to 85 °C. For experiments in which we incubated cells with enzyme inhibitors, 5 × 10^6^ cells were used. At least 3 independent biological replicates were conducted. Negative controls included beads-only or isotype IgG.

### Proteomics analysis

DIO3-eluted proteins were trypsinized and analyzed by LC-MS/MS on a Q Exactive™ HF-X Mass Spectrometer (Thermo Fisher Scientific, Bremen, Germany, outsourced at the Technion, Haifa, Israel). Proteins were identified by Discoverer software v1.4 against the human database using Sequest and Mascot. Peptides were filtered at 1% FDR with ≥ 2 unique peptides. Semi-quantification used the average peak area of the three most intense peptides per protein. Proteins were considered DIO3-associated if enriched > 4-fold versus beads-only and IgG isotype controls. Keratins were filtered out since they might be a contamination that origins from dust, hair and skin. The DIO3/Beads and DIO3/IgG ratio indicate enrichment of each SNX protein relative to background levels. Unique indicates detection only in the DIO3 pulldown.

### Protein enrichment analysis and classification

Protein classification was performed using http://www.geneontology.org/ powered by Protein Analysis through Evolutionary Relationships (PANTHER, http://pantherdb.org) [27]. Protein–protein interaction network was generated using STRING v. 11 (https://string-db.org) with default settings (minimum required interaction score: medium confidence 0.4) and clustered to a specified Markov clustering inflation parameter of 352 [28].

### Light microscopy

Images were obtained by an Olympus microscope equipped with a camera (model IX71 Olympus, Tokyo, Japan). Image analysis was performed by cellSens Entry Olympus imaging software.

### Immunofluorescence (IF) confocal microscopy

Cells were seeded for 24 h (1 × 10^5^ cells/24 well glass bottom plates), fixed for 10 min (4% PFA), permeabilized for 5 minutes (0.2% Triton X 100) and incubated with primary and secondary fluorescent antibodies. Nucleus was stained with Hoechst (33342, molecular probes, Eugene, OR, USA). Labeled secondary IgG antibodies were used as a negative control (Supplementary Fig. S1). For IF staining of FFPE tissues, we performed antigen retrieval (Ventana Benchmark XT automatic stainer), manual blocking (3% BSA), incubation overnight with selected antibodies and staining with secondary antibodies. Nucleus was stained with Hoechst 33,342 (Molecular probes, Eugene, OR, USA). Cells/tissues were analyzed by Leica SP5 confocal microscopy (Leica, Mannheim, Germany), using 20X/40X/63X objectives. A single 0.5 μm optical z-plane is shown in all figures. For subcellular localization, Z-stack images were acquired across the full cellular volume with optimal step size. Quantification was performed using the ImageJ (NIH) plugin Coloc2 (https://imagej.net/plugins/coloc-2), applying established metrics to avoid user bias.

### Proximity ligation assay (PLA)

Cells were seeded for 24 h (1 × 10^5^ cells/24 well glass bottom plates), fixed for 10 min (4% PFA), permeabilized for 5 minutes (0.2% Triton X 100). Proximity ligation assay was performed using the NavinciFlex Cell MR kit (Navinci, Uppsala, Sweden) and appropriate pairs of antibodies, following manufacturer’s instructions. Negative controls included omission of primary antibodies or isotype IgG (Supplementary Fig. S2). Red puncta indicate PLA signals (< 40 nm). To stain the nucleus, Hoechst 33,342 was used. 63X objective, scale bars: 15 μm. Quantifications of fluorescence intensity were performed using the NIH ImageJ software.

### Modeling of DIO3 and its interaction with SNX

A homodimeric model of the human DIO3 catalytic domain was generated via homology modeling [29], using the crystal structure of the monomeric mouse DIO3 catalytic domain as a template [23]. The resulting model was then superimposed onto peroxiredoxin dimer (poplar glutathione peroxidase, PDB ID: 2P5Q) and subsequently refined [30] to resolve steric clashes arising from the superimposition. We used the AlphaFold3 server [31] to predict the human DIO3 and protein complexes of DIO3 with-SNX2 (short and long isoforms), DIO3-SNX4, DIO3-SNX5 and DIO3-SNX6 (short and long isoforms). In this set of complexes, we used different DIO3 sequence templates as input: residues 68–304 for full cytosolic and catalytic domain regions, 110–304 for shorter cytosolic and full catalytic, and 120–304 and 127–304 for different catalytic domain lengths. To mitigate potential sequence order-related bias in AlphaFold3 predictions, each complex was modeled using both possible input orders – first with the SNX sequence followed by DIO3, and vice versa. For each complex, the model with the higher AlphaFold3 confidence score (Model 0) was selected to be the primary construct. For the primary construct - DIO3 110–304 sub-part in complex with SNX6 (short) - all ten AlphaFold3 output models were evaluated for solvent accessible surface area (SASA) to estimate the buried surface area upon complex formation [32]. The model exhibiting the largest SASA difference between complexed and uncomplexed forms was selected for subsequent interface analysis (Supplementary Fig. S3). AlphaFold3 includes four scores that measures model confidence: the predicted local-distance difference test (pLDDT), the predicted aligned error (PAE), the predicted template modeling (pTM) score and the interface predicted template modeling (ipTM) score. pLDDT quantifies the anticipated accuracy of each amino acid in the model relative to the real structure. Values are on a scale of 1 to 100 and the higher the score, the higher the confidence. PAE estimates the error in the relative position of the residues within the complex. Lower values indicate better confidence. The pTM and ipTM scores estimate the confidence in domain-level packing and inter-chain interactions, respectively [33, 34]. For pTM, a score above 0.5 indicates that the predicted model might be similar to the true structure. For ipTM, scores above 0.8 indicate highly confident predicted model, and scores between 0.6 and 0.8 may be inconclusive. Best-fit model was visualized using PyMOL [35]. Clustering of low pairwise PAE domains were visualized using ChimeraX 1.9 [36]. Maximum PAE value allowed between clusters was set to 5Å (default) and graph resolution (cluster) to 1.5.

### Statistical analysis

Data are presented as mean ± SEM of ≥ 3 independent experiments. Statistical significance was assessed by Student’s t-test or ANOVA (for multiple comparisons); *p* < 0.05 was considered significant. For proteomics, significant proteins were defined at *p* < 0.05 with FDR control [37].

## Results

### Sorting nexins are novel DIO3-associating proteins

**Fig. 1:**
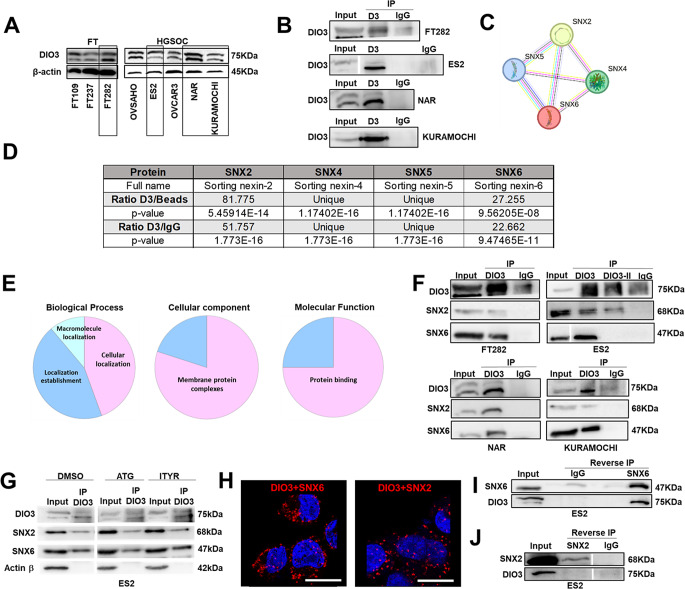
DIO3 associates with SNX family proteins. (**A**) DIO3 expression in a panel of human FT and HGSOC cell lines. Selected cells are marked by a black square (**B**) DIO3-IP in FT and three HGSOC cell lines (**C**) Protein-protein interaction network illustrating the interaction of SNX proteins using the STRING tool (**D**) SNX proteins identified following proteomic analysis of DIO3 Co-IP in ES-2 cells. The ratio was designated as unique when no assay background was observed (**E**) Functional classification of SNX proteins depicting biological process, cellular component, and molecular function classifications, using the PANTHER tool (**F**) WB analysis of DIO3, SNX2 and SNX6 following DIO3 Co-IP or IgG control from total protein extract of FT282, ES-2, NAR and KURAMOCHI cells. In ES-2 cells, a second elution was performed, annotated as DIO3-II. Skipped lanes are indicated by a separating line (Uncropped blots, Supplemental Fig. S7) (**G**) Co-IP results of DIO3, SNX2 and SNX6 from lysates of ES-2 cells cultured overnight with DMSO, ATG or ITYR-DBRMD. β actin, was used as a negative control. Results were repeated at least twice. (**H**) PLA of DIO3 and SNX2 or SNX6 in ES-2 cells. WB analysis of reverse Co-IP using antibodies against (**I**) SNX6 or (**J**) SNX2 in protein extracts from ES-2 cells. Skipped lanes are indicated by a separating line (Uncropped blots are presented at Supplementary Fig. S7)

DIO3 is recycled bidirectionally between the plasma membrane and early endosomes [9]. However, the exact mechanism facilitating this trafficking is still largely unknown. In this work, we investigated DIO3 association with sorting nexins in HGSOC cells by several complementary methods, including DIO3 Co-IP. Currently no DIO3 antibody was validated for this specific application, therefore we optimized an antibody which recognizes both the monomeric (~ 37 kDa) and the active DIO3 homodimer (~ 75 kDa) forms [5], following a similar protocol as we recently published for another protein [26]. COS7 cells overexpressing mouse DIO3-Flag [23] were used to validate antibody specificity (Supplementary Fig. S4). Human immortalized fallopian tube cells (FT282) as well as HGSOC lines, ES-2, NAR and KURAMOCHI cells, expressing high levels of endogenous DIO3 dimers, were selected for performing the Co-IP studies in human HGSOC cell lines (Fig. 1A). Total protein lysates from the different cell lines were extracted and effective DIO3 pull-down of the DIO3 dimer was confirmed with low background, using a negative IgG isotype antibody (Fig. 1B). This series of experiments substantiated the specificity and validity of the DIO3 antibody for IP applications.

The successful elution of DIO3 allowed us to assess potential DIO3-bound proteins by performing DIO3 Co-IP in ES-2 cells, serving as the proof-of-concept model. Protein lysates from two independent repeats underwent LC-MS/MS proteomics analysis. Proteins were considered potential DIO3-interacting partners when greater than 4-fold binding was calculated using the specific DIO3 antibody, relative to Co-IP with beads only or IgG (negative controls). In addition, only significant proteins following FDR (*p* < 0.05) were included. Among several novel proteins bound to DIO3, we identified four SNXs, including SNX2, SNX4, SNX5 and SNX6, which belong to a sub family of SNXs that contains PX and BAR domains [12, 13] and show high sequence similarity (Supplementary Fig. S5). STRING analyses (Fig. 1C) illustrate an interaction between these four SNXs, with a highly significant protein-protein interaction (PPI) enrichment p value (1.05e-11), confirming a biological connection. Quantitative enrichment levels of the various SNX proteins in the DIO3 immunoprecipitate with a high DIO3/Beads and DIO3/IgG ratio (Fig. 1D), reflect a potentially strong association. Functional classification by PANTHER analyses shows that these SNXs are predominantly linked to biological processes involving cellular localization, membrane-associated complexes and protein-binding activities, supporting their function in vesicle-mediated transport (Fig. 1E). As the four identified sorting nexins belong to the same SNX-BAR subfamily [14], we decided to explore DIO3 association with SNX2 and SNX6. First, we performed DIO3 Co-IP in fallopian tube cells (FT282) as well as HGSOC cell lines (ES-2, NAR and KURAMOCHI). We confirmed that DIO3 elutes with both sorting nexins in the various models (Fig. 1F), showing a higher association with SNX6 in comparison to that observed with SNX2. As ES-2 cells displayed superior expression of DIO3 and SNXs, these cells served as the proof-of-concept model for further studies.

We next explored whether DIO3 enzymatic activity is essential for its interaction with SNX2 and SNX6. For that, we incubated ES-2 cells overnight with a non-specific deiodinase inhibitor (ATG, 50µM) which inactivates all deiodinase family members (DIO1, DIO2 and DIO3) or with a DIO3-specific inhibitor (ITYR-DBRMD, 500µM) which we recently developed [6]. Under these experimental settings the drugs are expected to inhibit the enzyme catalytic activity without affecting the cell phenotype. DMSO, in which the drugs are dissolved, was used as vehicle control. Total protein lysates from the various treatments underwent Co-IP using a DIO3 antibody and WB were prepared from the eluted proteins. Results indicate that both SNX2 and SNX6 remain in complex with DIO3 (Fig. 1G), suggesting that this association does not require DIO3 enzymatic activity. Similarly, DIO3 silencing did not affect SNX2 or SNX6 protein expressions (Supplementary Fig. S6).

The protein-protein relations between DIO3-SNXs were next confirmed by Proximity Ligation Assay (PLA), which produces fluorescence signal for protein pairs within less than 40 nm, reinforcing these associations in situ (Fig. 1H). These collective results substantiate that DIO3 associates with SNX6 and SNX2 in ovarian cancer. Reverse Co-IP for SNX6 confirmed that the link with DIO3 is bidirectional (Fig. 1I), while SNX2 did not elute DIO3 (Fig. 1J), which may reflect weaker assocication levels.

### DIO3 colocalizes with SNX2 and SNX6 in the cytosol of normal or premalignant fallopian tubes and tumors from HGSOC patients

**Fig. 2:**
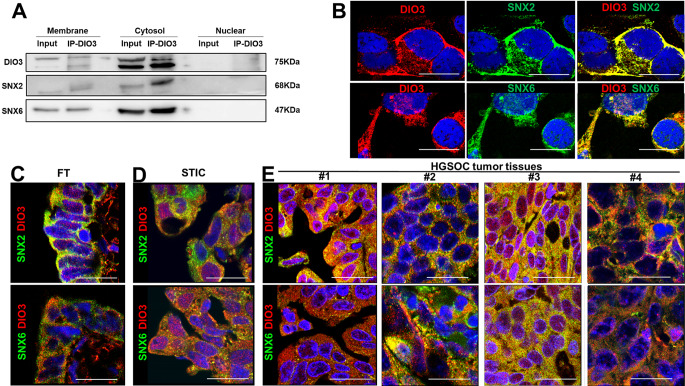
DIO3 colocalizes with SNX2 and SNX6 in ovarian cancer cells and human tissues. (**A**) WB analysis of SNX2 and SNX6 following Co-IP using anti DIO3 antibody in membrane, cytosol and nuclear protein extracts of ES-2 cells (**B**) ES-2 cells were seeded in glass bottom 24-well plates and stained far red for DIO3 and green for SNX2/SNX6. FFPE tissues from (**C**) normal FT (**D**) premalignant STIC lesions and (**E**) four HGSOC tumors from human patients were stained for SNX2/SNX6 (green) and DIO3 (red). In all images, a single 0.5 μm optical section using 63X objective is presented using Leica SP5 confocal microscopy. Scale bars: 20 μm. Separate channels are shown on Supplementary Fig. S9

Following the observation that SNX2 and SNX6 associate with DIO3, we aimed to uncover the exact site of this colocalization. To that end, we isolated protein fractions from the membrane, cytosol and nucleus of ES-2 cells and performed DIO3 Co-IP. Results indicate that DIO3 colocalizes with SNX2 and SNX6 primarily in the cytosol (Fig. 2A), reflecting intracellular membrane-bound compartments within the cytoplasm. Purity was validated using fraction markers (Supplementary Fig. S8). Confocal immunofluorescence (IF) microscopy confirmed a similar colocalization between DIO3 (red) and SNXs (green) (Fig. 2B).

To assess the localization of DIO3-SNXs in clinical settings, confocal IF microscopy was performed on tissues from human patients including normal fallopian tube, premalignant serous tubal intraepithelial carcinoma (STIC) and four HGSOC tumors. A well-structured DIO3-SNXs merging was documented in normal fallopian tube cells (Fig. 2C). In premalignant STIC lesions (Fig. 2D), and more so in tumor tissues (Fig. 2E), colocalization between DIO3 and SNXs was greatly enhanced in the cytosol. These findings suggest that DIO3 trafficking may relate to SNX2 and SNX6 in both normal and malignant tissues.

### Computational prediction of DIO3 association with SNX2 or SNX6

**Fig. 3:**
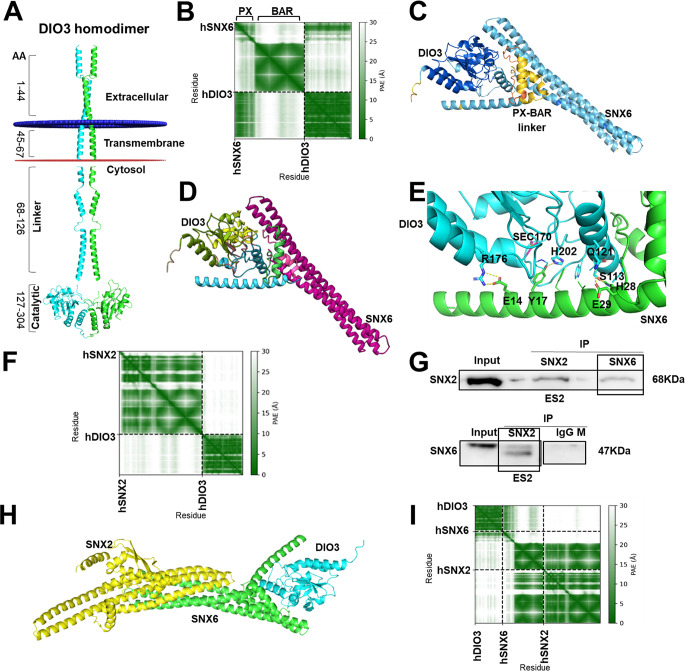
Computational modeling of DIO3 interaction with SNX6 and SNX2. (**A**) Predicted human DIO3 (hDIO3) dimer by AlphaFold3 for the extracellular and linker regions and by homology modeling for the catalytic region, represented as cartoon and chain colored. The membrane boundaries are shown as horizontal planes, colored blue and red (**B**) predicted aligned error (PAE) heatmap of the interface between DIO3 (110–304) and the short human SNX6 (hSNX6) isoform, using AlphaFold3. The PX and BAR domains in SNX6 are marked (**C**) The DIO3-SNX6 complex represented as cartoon and colored based on Alphafold3 predicted Local Distance Difference Test score (pLDDT) as follows: Blue - Very high confidence > 90, cyan –between 70 and 90, yellow – low, between 50 and 70, orange - very low < 50. (**D**) The predicted DIO3-SNX6 complex represented as cartoon and clustered into coherent domains of relatively low pairwise PAE values, using ChimeraX 1.9 (**E**) Magnified view of the predicted DIO3-SNX6 interface. DIO3 and SNX6 are represented in cartoons, colored cyan and green respectively. Residues involved in polar interactions are represented as sticks, element-colored (carbon-cyan/green, red-oxygen, blue-nitrogen, yellow-sulfur) and labeled. The polar interactions are shown in a yellow dashed line. Residues involved in hydrophobic interactions are represented as lines and element colored. Active site residue (Cys170) is represented as sticks and element-colored magenta (**F**) PAE heatmap of the DIO3 (110–304) and the human short SNX2 isoform (hSNX2) complex (**G**) WB analyses of Co-IP in ES-2 cells using SNX2 and SNX6 antibodies, confirming co-elution of both proteins. Input lanes represent total protein lysate and IgG lanes served as negative control (**H)** SNX6-SNX2-DIO3(110–304) in complex, represented as cartoon and colored green, yellow and cyan respectively (**I**) PAE heatmap of DIO3 (110–304), SNX2 and SNX6 hetero-trimeric complex

Parallel to empirical validation that DIO3 is associated with SNX2 and SNX6, we were interested in examining whether this collaboration could be predicted by computational models using AlphaFold3 [31]. The full length of the human DIO3 (Fig. 3A) contains an extracellular N-terminal region (residues 1–44), transmembrane domain (residues 45–67), cytosolic C-terminal domain which includes a linker region (residues 68–126) and the catalytic domain (residues 127–304) [23]. For performing predictions of DIO3 complexing with SNX2 and SNX6, we focused on the DIO3 cytoplasm-exposed regions. We analyzed five DIO3 sub-parts, including full linker and catalytic site (residues 68–304), full linker with a short fragment of the catalytic site (residues 68–126), short linker region and catalytic site (residues 110–304), full catalytic domain (residues 120–304), and shorter catalytic domain (residues 127–304). Interface confidence scores of each of the DIO3 structures were modeled with the short and long SNX2 and SNX6 isoforms (Supplementary Fig. S10). Results revealed that the short isoform of SNX6 yielded relatively confident interaction models with both the 68–304 and 110–304 DIO3 sub-parts, with the latter showing slightly higher interface confidence score (Supplementary Table S2). A similar result was obtained by PAE heatmap analysis (Supplementary Fig. S10). This suggests that both the catalytic and linker regions of DIO3 are essential for interaction with SNX6. In detail, the DIO3 110–304 complex with the short SNX6 isoform showed low PAE values ( < ~ 3–5 Å) in DIO3, in the PX and BAR domains of SNX6, and in the intersection between DIO3 and the PX domain, indicating higher structural consistency within the model (Fig. 3B). However, the relative orientation of the PX domain with respect to the BAR domain in SNX6 remains uncertain. This was evident by the elevated PAE values (PAE > 20 Å) in the intersection between the PX domain and the BAR domain in the PAE heatmap. Additionally, the local confidence scores of the PX-BAR linker show lower confidence (colored yellow and orange, pLDDT < 70, 50 respectively) (Fig. 3C). Notably, the interface between DIO3 and a short segment of the PX domain exhibited a high local confidence for both proteins individually (colored cyan and blue pLDDT > 70, 90 respectively), supporting the plausibility of a specific connection between SNX6 and DIO3. Taken together, AlphaFold3 is unable to predict reliably the position of the PX domain in relation to the BAR domain in SNX6. Despite that uncertainty, AlphaFold3 prediction suggests a potential interaction between DIO3 and the PX domain.

To further investigate this, continuous low-pairwaise PAE color-clustering was performed, thereby delineating related domains within the complex (Fig. 3D). This analysis identified distinct PAE-based domains, among which only one (colored cyan) spanned both DIO3 and the PX domain of SNX6, signifying a potential interface between them. The resulting DIO3-SNX6 interface suggests contact region between residues ~ 14–30 of SNX6 and to the DIO3 linker (residues ~ 110–120) and regions neighboring the catalytic Cys170 (SeCys170) (Fig. 3E). Moreover, analysis of the predicted DIO3-SNX6 interface showed several residues that could be involved in the stabilization of the interface. Four residues in SNX6 were at a sufficient distance from their corresponding partners in DIO3 to form polar interactions (E14SNX6-R176DIO3, Y17SNX6-H202DIO3, H28SNX6-S113DIO3, and E29SNX6-Q121DIO3). There is also a notable hydrophobic interaction network contributed by the benzene, phenol, pyrrolidine indole and imidazole rings (F21SNX6, H202DIO3, Y17SNX6, W207DIO3 and P277DIO3, respectively) and Valines (V25SNX6-V117DIO3). Future mutational and truncation studies would be valuable for fine mapping of the specific interaction interfaces. In this predicted interface, the catalytic C170 is pointing into the interface and is unable to react. Nevertheless, C170 is located near the loop-D (or omega-loop) that is critical to the iodothyronine-binding and is not obstructed by SNX6. Unlike SNX6, SNX2 connection with DIO3 did not produce high-confidence PAE values (Fig. 3F). It is well known that SNXs with BAR domains such as SNX2 and SNX6 can form heterodimers [12, 13]. We validate that in our ovarian cancer cells this heterodimer is present (Fig. 3G). This, together with our results that DIO3 is eluted by SNX6 but not by SNX2 antibodies (Fig. 1I), motivated us to model the SNX2-SNX6 heterodimer interface with DIO3 (Fig. 3H). This model showed a high ipTM: pTM score (Fig. 3I and Supplementary Table S2) suggesting that SNX2-DIO3 association may be mediated by SNX6.

### SNX2 and SNX6 are associated with DIO3 trafficking through the endosomal network

**Fig. 4:**
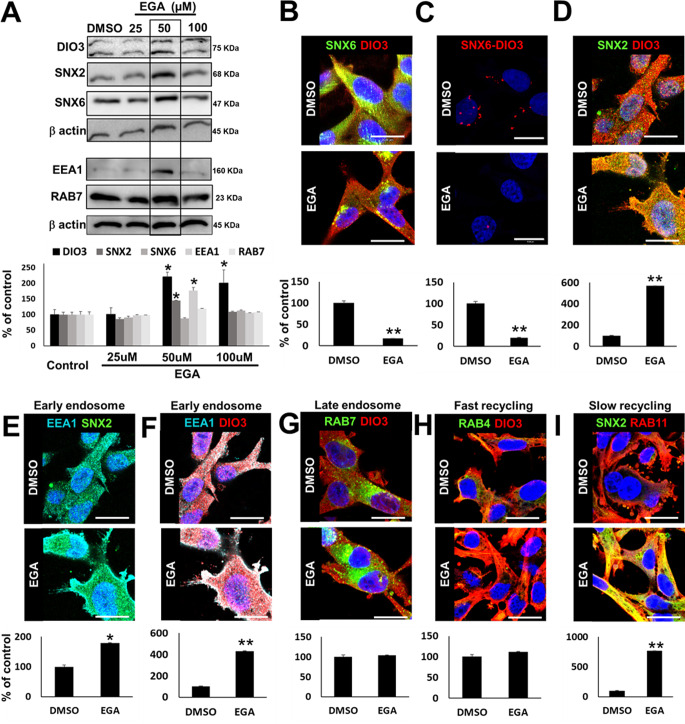
SNX2 and SNX6 are distributed with DIO3 trafficking through the endosome network. (**A**) WB analysis of DIO3, SNX2, SNX6, EEA1 and RAB7 following treatment with EGA at various concentrations. Representative IF confocal microscopy images of ES-2 cells treated with EGA for 3 h showing (**B**) DIO3 (red) and SNX6 (green) (**C**) Proximity Ligation Assay (PLA) of DIO3 and SNX6 (**D**) DIO3 (red) colocalization with SNX2 (green) (**E**) EEA1 (far red) with SNX2 (green). DIO3 (red) with (**F**) EEA1 (far red) (**G**) RAB7 (green) (**H**) RAB4 (green) (**I**) SNX2 (green) and RAB11 (red). 63X objective. Scale bars: 20 μm (IF) and 15 μm (PLA). Supplementary Figure S11 depicts separate channels for all confocal images. Quantifications of merged channels for each proteins pairs are presented as % of control below each figure. Experiments were repeated twice, in duplicates. *, *p* < 0.05. **, *p* < 0.005

The novel links between SNX2 and SNX6 with DIO3, together with previous reports that DIO3 is recycled via endosomes [9], led us to examine which of these sorting nexins is distributed with DIO3 trafficking through the endosomal compartments. One approach could be by silencing or knocking out SNX2 or SNX6. However, sorting nexins regulate trafficking of numerous proteins and would cause broad, multimodal changes with extensive functional consequences that are not specific to DIO3 [38–41]. Therefore, SNX silencing is an unsuitable platform for understanding DIO3-SNX association. Instead, we explored the overlap of DIO3 with SNX2 or SNX6 by using several specific inhibitors for the transition between endosomes. First, we treated ES-2 cells with EGA, which blocks the transition from early to late endosome, without affecting recycling endosome trafficking [42]. 50µM EGA, which demonstrated a decrease in SNX6 and a significant increase in DIO3, SNX2 and EEA1, an early endosome marker, was selected for further studies (Fig. 4A). Results indicate that colocalization of SNX6 with DIO3 was reduced by 84% upon EGA treatment and seem to concentrate at limited regions adjacent to the nucleus, as shown by IF confocal microscopy (Fig. 4B). We speculate that such perinuclear clustering reflects retromer-mediated transport to the Golgi apparatus [19, 20]. PLA analyses confirmed a comparable 80% reduction in DIO3-SNX6 association (Fig. 4C). These results suggest that SNX6 may be required for trafficking of DIO3 from early to late endosome. In contrast, under EGA treatment, DIO3 overlaps with SNX2 was significantly enhanced (Figs. 4D and 5.7-fold, *p* = 0.004). This colocalization is most likely in early endosomes, as demonstrated by a parallel induction in the overlap of EEA1 with both SNX2 (Fig. 4E, 1.8-fold, *p* = 0.01) and DIO3 (Fig. 4F, 4.3-fold, *p* = 0.005), but not with the late endosome marker RAB7 (Fig. 4G). Furthermore, while DIO3 was not distributed together with the fast-recycling endosome marker RAB4 (Fig. 4H), SNX2 exhibited considerable merging with RAB11, a marker of recycling endosome (Figs. 4I and 7.7-fold, *p* = 0.003). These collective results support preferential transport from early endosomes to the plasma membrane via a controlled, slow-recycling process, potentially associated with SNX2.

### Trafficking of DIO3 via the ER-Golgi colocalizes with SNX6

**Fig. 5:**
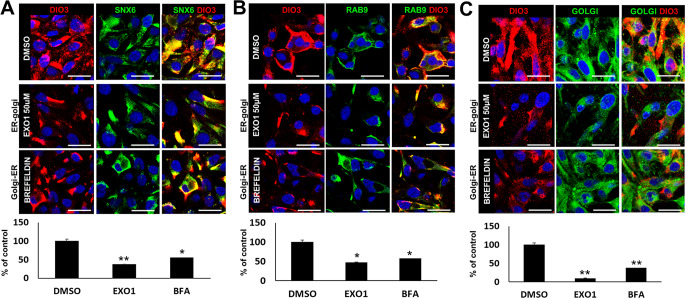
ER to Golgi and retrograde transport of DIO3 is distributed with SNX6. IF confocal microscopy analysis of DIO3 (red) and (**A**) SNX6 (green) (**B**) RAB9 (green) and (**C**) Golgi apparatus staining (green) in ES-2 cells treated for 3 h with EXO1 (50µM) or BFA (3 µg/ml). 63X objective. Scale bars: 20 μm. Quantifications of merged channels for each proteins pairs are presented as % of control below each figure. Experiments were repeated twice, in duplicates. *, *p* < 0.05. **, *p* < 0.005

We observed that SNX6 is distributed in perinuclear regions consistent with vesicular trafficking pathways involving DIO3, in proximity to the Golgi apparatus. To further assess the involvement of Golgi-associated trafficking, we utilized EXO1, an inhibitor of ER-to-Golgi transport [43]. Upon EXO1 treatment, spatial overlap of DIO3 with SNX6 (Fig. 5A, middle panel) and with RAB9 (Fig. 5B, middle panel), a marker of late endosomal compartments functionally linked to the trans-Golgi network was reduced by 63% and 53%, respectively. In addition, a significant 91% reduction was observed in DIO3 overlap with a Golgi-selective fluorescent probe that preferentially stains trans-Golgi stacks (Fig. 5C, middle panel). These findings indicate that DIO3 localization to Golgi-associated and endosomal compartments may be linked to SNX6-dependent trafficking.

We further used BFA, which inhibits transport from the Golgi to the ER [43], in order to study whether DIO3 undergoes retrograde transport between these two organelles and to explore SNX6 involvement. Results indicate that the association of SNX6 with DIO3 in this retrograde transport was reduced by 45% when this trafficking is hindered (Fig. 5A, lower panel). This was accompanied by a parallel 43% reduction in DIO3 overlay with RAB9 (Fig. 5B, lower panel) and a 63% reduction in its association with Golgi staining (Fig. 5C, lower panel). SNX6 loss of colocalization with DIO3 under EXO1 and BFA, together with reduced overlap with RAB9 and a Golgi marker, support a role for intracellular trafficking in regulating DIO3 localization and its association with SNX6, rather than a specific involvement of SNX6 in Golgi-to-ER trafficking.

### SNX2 and SNX6 are distributed with DIO3 during endocytosis and membrane recycling

**Fig. 6:**
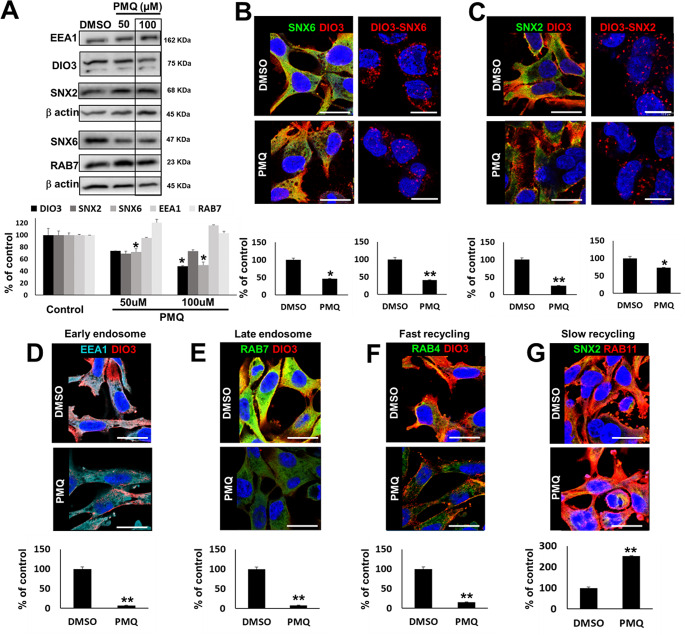
DIO3 endocytosis and membrane recycling colocalizes with SNX2 and SNX6 (**A**) WB analysis of DIO3/SNX2/SNX6/EEA1/RAB7 under PMQ in ES-2 cells. Representative IF confocal microscopy images of ES-2 cells treated with 100 µM PMQ (3 h, showing (**B**) Left panel-DIO3 (red) and SNX6 (green), right panel -PLA analyses (**C**) Left panel-DIO3 (red) colocalization with SNX2 (green), right panel - PLA analyses. DIO3 (red) with (**D**) EEA1 (far red) (**E**) RAB7 (green) and (**F**) RAB4 (green) (**G**) SNX2 (green) and RAB11 (red). 63X objective. Scale bar: 20 μm (IF) and 15 μm (PLA). Supplementary Fig. S12 depicts separate channels. Quantifications of merged channels for each proteins pairs are presented as % of control below each figure. Experiments were repeated twice, in duplicates. *, *p* < 0.05. **, *p* < 0.005

To substantiate that SNX2 and SNX6 may be associated with recycling of DIO3 to the membrane, we utilized Primaquine (PMQ), which disrupts recycling endosome function, blocking reusing of membrane proteins [44]. 100 µM PMQ led to a reduction in the protein levels of DIO3, SNX2 and SNX6 and was selected for further studies (Fig. 6A). Disturbing membrane recycling resulted in a 54–60% decreased association of DIO3 with SNX6, by confocal and PLA analyses (Fig. 6B). Confocal IF microscopy further indicated a 75% reduction in DIO3 merging with SNX2, while PLA demonstrated a 27% decrease (Fig. 6C). Moreover, blocking recycling back to the membrane led to a significant ~ 90% decrease in the expression of DIO3 in early (Fig. 6D), late (Fig. 6E) and fast-recycling (Fig. 6F) endosomes. Therefore, hindering DIO3 recycling back to the membrane leads to a cascade of impaired intracellular endosome maturation. Concurrently, the significant increase in RAB11 (Fig. 6G), alongside the decrease in SNX2, suggests slow-recycling endosome accumulation and disrupted SNX2-related sorting.

**Fig. 7:**
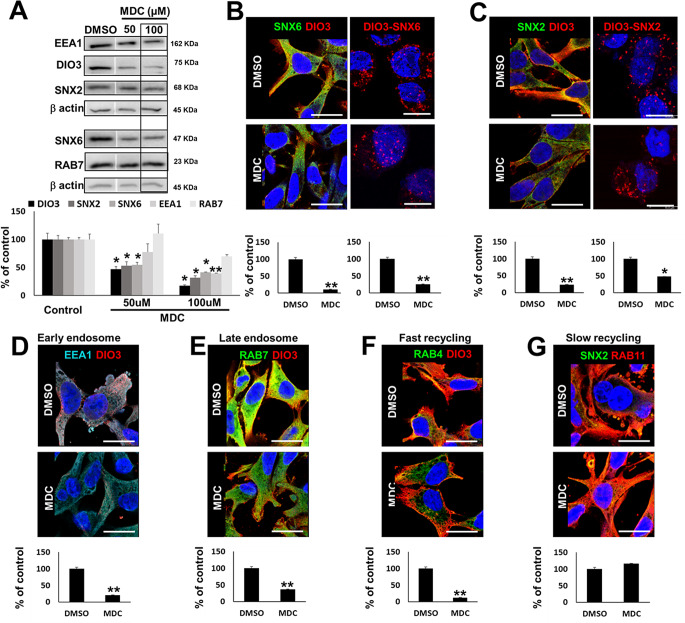
SNX6-SNX2 are distributed with DIO3 during endocytosis. (**A**) WB of DIO3/SNX2/ SNX6/ EEA1/RAB7 under MDC in ES-2 cells. Skipped lanes are marked (Uncropped blots, Supplemental Fig. S13). Confocal microscopy images of cells under 100 µM MDC for 3 h showing (**B**) Left panel-DIO3 (red) and SNX6 (green), right panel-PLA analyses. (**C**) Left panel-DIO3 (red) merging with SNX2 (green), right panel-PLA analyses. Confocal IF microscopy of DIO3 (red) and (**D**) EEA1 (far red) (**E**) RAB7 (green) and (**F**) RAB4 (green) (**G**) SNX2 (green)/RAB11 (red). 63X objective. Scale bars: 20 μm (IF) and 15 μm (PLA). Supplemental Fig. S14 depicts separate channels for all confocal images. Quantifications of merged channels for each proteins pairs are presented as % of control below each figure. Experiments were repeated twice, in duplicates. *, *p* < 0.05. **, *p* < 0.005

As DIO3 was reported to enter the cell via clathrin-dependent endocytosis [9], we incubated ovarian cancer cells with Monodansylcadaverine (MDC), an inhibitor of clathrin mediated entry [45]. Like previous inhibitors, an optimal 100 µM concentration was selected based on WB analysis showing a reduction in DIO3, sorting nexins and endosome markers (Fig. 7A). Under MDC, as demonstrated by IF and PLA confocal microscopy, DIO3 expression and colocalization with SNX6 (Fig. 7B) was significantly reduced by 90% and 75%, respectively. Likewise, a reduction of 77% and 53% was observed for DIO3-SNX2 association, respectively (Fig. 7C). Preventing clathrin mediated endocytosis was accompanied by a 79% DIO3 reduction in early endosome (Fig. 7D), 64% in late endosome (Fig. 7E) and 88% in fast-recycling endosome (Fig. 7F), while slow-recycling endosome remained unaffected (Fig. 7G). These collective results suggest association of both SNX2 and SNX6 in DIO3 internalization from the cell membrane and corroborate that the dynamics of DIO3 require precise trafficking through all endosomal compartments.

### Perturbing DIO3 trafficking has cellular consequences

We have recently reported that DIO3 expression is functionally connected with pro-tumorigenic activities in ovarian cancer and that silencing or inhibiting this enzyme has anti-cancer effects in ovarian cancer cells and animal models [5, 6, 46]. Here, we sought to explore whether disruption of DIO3 trafficking is not only structural, but also impairs DIO3-dependent cellular effects, linking intracellular routing with DIO3 function.

**Fig. 8:**
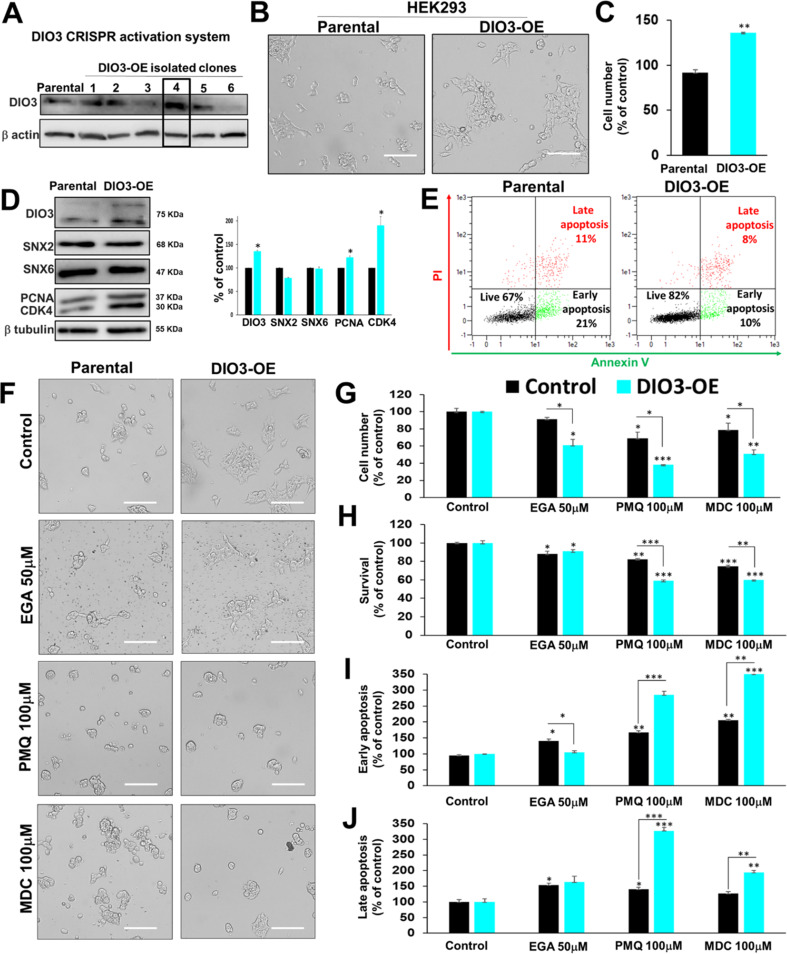
Perturbing DIO3 trafficking leads to lethal cellular consequences. DIO3 overexpression (DIO3-OE) was produced by CRISPR activation system in HEK293 cells (**A**) WB validation of DIO3-OE isolated clones (1–6). Clone #4 was chosen. β actin, loading control (**B**) Representative bright-field images of parental vs. DIO3-OE cells. 10X objective. Scale bars: 100 μm (**C**) Quantification of cell number, % of control, flow-cytometry (**D**) DIO3, SNX2, SNX6, PCNA and CDK4 protein levels in parental vs. DIO3-OE cells. β tubulin, loading control. Normalized bands quantifications (% of control), right panel (**E**) Annexin V/PI staining showing apoptosis distribution in parental vs. DIO3-OE cells (**F**) Microscopy images of parental and DIO3-OE cells treated with vesicle-trafficking inhibitors (EGA, PMQ, MDC). The same cells were analyzed for (**G**) cell number (**H-J**) Annexin V/PI, for surviving, early apoptosis or late apoptosis cell fraction. Experiments were repeated twice, in triplicates. Data are presented as % of control, *p < 0.05, **p < 0.005, ***p < 0.0005

We decided to overexpress DIO3 in HEK293 cells, which display basal levels of this enzyme. This cell platform provides high transfection efficiency and stable growth, enabling mechanistic isolation of DIO3-dependent effects. Cells were transfected using the DIO3 SAM-CRISPR activation system (DIO3-OE), while a non-specific CRISPR activation plasmid was used as negative control (parental). Following antibiotics selection and clonal isolation, clone #4 was chosen for further studies (Fig. 8A). DIO3-OE increased cell density (Fig. 8B) cell number (Fig. 8C) and the expression of the cell proliferation proteins, PCNA and CDK4, while maintaining SNX2/SNX6 signature (Fig. 8D). In parallel, reduced apoptotic priming was observed (Fig. 8E). Collectively, these results, which are comparable with the established pro-mitogenic activity of DIO3 in ovarian cancer models [5, 6], validate our DIO3 CRISPR activation system as a robust and reproducible tool. This cellular platform was then used for probing DIO3 function and its downstream trafficking-dependent pathways, by pharmacologically perturbing endosomal trafficking, using EGA, PMQ or MDC or vehicle control (DMSO), similar to the experimental settings in HGSOC cells. In comparison to parental cells, DIO3-OE cells became selectively vulnerable to all three inhibitors with reduced cell density (Fig. 8F), cell number (Fig. 8G) and cell survival (Fig. 8H), with a parallel elevation in early (Fig. 8I) and late apoptosis (Fig. 8J). These data indicate that the intracellular itinerary of DIO3 through the endosomal route is not a passive trafficking event but an obligate requirement that enables the pro-survival/pro-proliferative phenotype of DIO3.

## Discussion

DIO3 is a membrane integral protein which loses its enzymatic activity upon purification [47]. This loss of activity was attributed to disruption of essential phospholipid-protein interaction [21]. DIO3 is active as a homodimer that traffics to and from the plasma membrane. It undergoes endocytosis into early endosomes, a process which is required for reactivation of the enzyme catalytic site, most probably by thiol cofactor [9, 47]. The majority of DIO3 is not retained in the endosome for lysosomal degradation but is exported back to the cell membrane for reuse, explaining the enzyme long half-life [9]. This localization of DIO3 in endosomes was reported in neuronal cells and monkey hepatocarcinoma cells [9–11]. In this work, however, we provide the first observation of DIO3 localization in the endosomal network of human healthy fallopian tubes, as well as premalignant and malignant ovarian cancer cells and tissues, in which a pro-mitogenic role for this enzyme was demonstrated [5, 6].

Despite its well-documented enzymatic function and its increasingly appreciated role in promoting tumorigenesis, DIO3 trafficking inside the cell has remained elusive. In this work we provide the first insight into the molecular chaperones that may enable this recycling. Here, we present evidence that DIO3 associates with specific members of the sorting nexin protein family, particularly SNX2 and SNX6, and traffics through the endosomal and Golgi networks. These findings stem from an integrative approach combining the utilization of an array of trafficking inhibitors, proteomics, Co-IP, advanced confocal imaging, and computational protein structure modeling. Our results show that DIO3 colocalizes with SNX2 and SNX6 in premalignant and ovarian cancer cells and patient-derived tissues, occurring mainly in the cytosolic fraction. These observations align with the fact that SNX2 and SNX6 are PX domain-containing proteins that dynamically cycle between the cytosol and PI3P-enriched endosomal membranes. Similarly, DIO3 undergoes continuous endocytic trafficking, supporting a transient and dynamic intracellular cytosolic distribution, rather than stable confinement to bulk membrane compartments [9]. Healthy fallopian tubes considered the disease site of origin [7, 8], displayed lower levels of DIO3-SNX co-distributions.

To complement our experimental data, we utilized AlphaFold3 predictions to suggest a potential structural basis for the link between DIO3 and SNX2 or SNX6. While most of the predicted models were assigned low-confidence scores, the DIO3 110–304 construct revealed a high-confidence interface between the short isoform of SNX6 and DIO3. The predicted model falls within the upper end of the AlphaFold3’s “gray zone” boundaries, that indicates a possibility for a structural uncertainty. However, the recurrence of similar DIO3 110–304–SNX6 interfaces across all varying conformation complex models supports the plausibility of this interface. Additionally, the low confidence between the PX and BAR domains in SNX6 may contribute to the variability in model confidence yet does not preclude the existence of this specific interface model. This interaction is predicted to involve the PX domain of SNX6 [12, 13] and regions of DIO3 adjacent to its catalytic site. DIO3 lacking either the catalytic core or the linker region, showed no predicted association with SNX6, underscoring the necessity of both elements for complex formation. While the catalytic cysteine (C170) appears buried in the modeled interface, the proximity of SNX6 to key substrate recognition loops suggests a regulatory rather than enzymatic interaction. DIO3 remained in complex with both SNXs under DIO3 inhibition, suggesting that this association may not require DIO3 enzymatic activity. These computational predictions reinforce the biochemical data and propose a specific docking pose for DIO3 within SNX6-containing endosomal carriers. Notably, the SNX2-SNX6 heterodimer modeled with DIO3 demonstrated a high-confidence score. These two sorting nexins are known to form heterodimers through a conserved BAR domain [12–17] and to play a critical role in endosome–to–plasma membrane recycling and retromer transport to the trans-Golgi [18–20, 48].

Disruption of trafficking pathways with pharmacological inhibitors allowed us to temporally dissect the transport steps involving DIO3 and SNXs. Blocking early-to-late endosome maturation reduced DIO3–SNX6 colocalization and increased DIO3–SNX2 merging, reflecting arrest in early endosomes. Conversely, the inhibition of recycling impaired the association with SNX2 without disrupting SNX6. This dichotomy supports a model in which SNX6 and SNX2 operate in separate yet coordinated arms of the DIO3 trafficking route. Besides trafficking through endosomal routing, we provide indications that DIO3 is transported between the ER and trans-Golgi organelles, as well as retrograde transition from endosomes to the Golgi. This suggests that DIO3 shuttling spans multiple compartments involved in protein sorting and processing. We further established that this trafficking colocalizes with SNX6. This dual functionality echoes the potential broader role of SNX6 in cargo retrieval from late endosomes and Golgi compartments. Additionally, this study provides, for the first time in ovarian cancer, data on the expression patterns of SNX2 and SNX6, for which studies suggested potential roles in modulating other neoplastic diseases [22, 49–53]. However, although this study identifies association, colocalization, and pathway sensitivity of DIO3, additional experiments are merited for concluding a direct mechanistic causality.

**Fig. 9:**
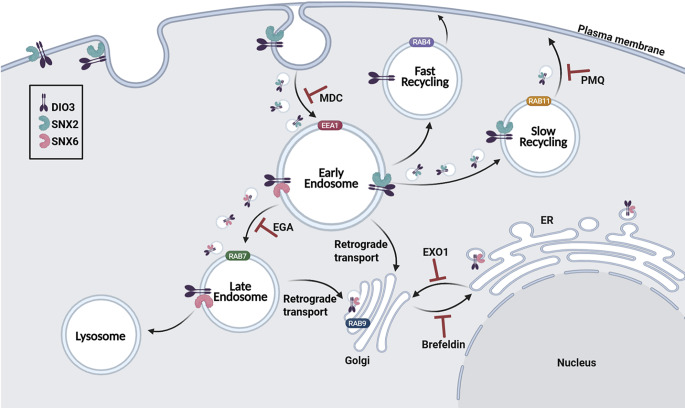
Proposed model of DIO3 trafficking controlled by SNX2/SNX6

Using a DIO3-overexpression cell platform we further established that its trafficking through the endosomal network is functionally dependent, and disrupting this route exposes a discrete vulnerability of DIO3-high cells. A summary of DIO3 suggested trafficking routes is presented in Fig. 9.

In conclusion, our findings propose a novel SNX-associated trafficking of DIO3 in ovarian cancer. This compartmentalized transport system allows for dynamic regulation of DIO3 localization and may represent a new layer of control for the enzyme bioavailability. Our study provides new insights regarding the biological consequences of altered DIO3 trafficking, with potential implications in physiological conditions and other diseases, beyond ovarian cancer.

## Supplementary Information

Below is the link to the electronic supplementary material.

Supplementary Material 1

Supplementary Material 2

## Data Availability

Full proteomics datasets are available from the corresponding author upon reasonable request.

## Abbreviations

BAR: Bin/Amphiphysin/Rvs domain
BFA: Brefeldin A
Co-IP: Co-immunoprecipitation
DIO3: Type 3 iodothyronine deiodinase
ER: Endoplasmic reticulum
HGSOC: High-grade serous ovarian cancer
ipTM: Interface predicted template modeling
MDC: Monodansylcadaverine
PLA: Proximity ligation assay
PX: Phox homology
PMQ: Primaquine phosphate
pLDDT: Predicted local-distance difference test
PAE: Predicted aligned error
pTM: Predicted template modeling
PPI: Protein-protein interaction
SNX: Sorting nexin
STIC: Serous tubal intraepithelial carcinoma
