# Supplementary material for: DIO3 associates with sorting nexins and endosomal trafficking networks in ovarian cancer
Source: Cell Oncol (Dordr). 2026 Jun 2;49(4):104. doi: 10.1007/s13402-026-01236-z (PMC13447639; doi:10.1007/s13402-026-01236-z)
Supplement: Supplementary file 1 [file 13402_2026_1236_MOESM1_ESM.pdf]

Supplementary Figures

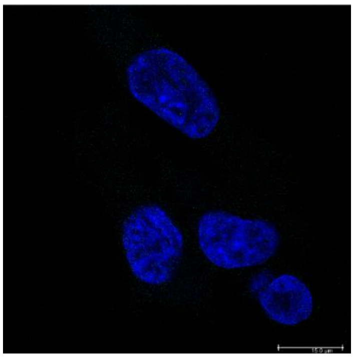

Supplementary Figure S1. Negative control for IF confocal microscopy images. ES2 cells were stained using secondary labeled antibodies. The nucleus was stained with Hoechst 33342.

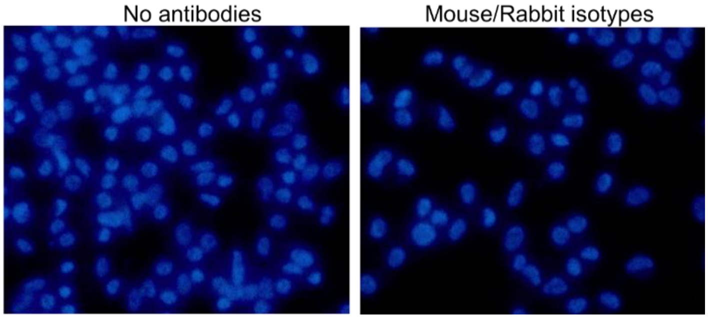

Supplementary Figure S2. Negative control for PLA. ES2 cells were stained using Mouse and Rabbit isotype control antibodies. The nucleus was stained with Hoechst 33342.

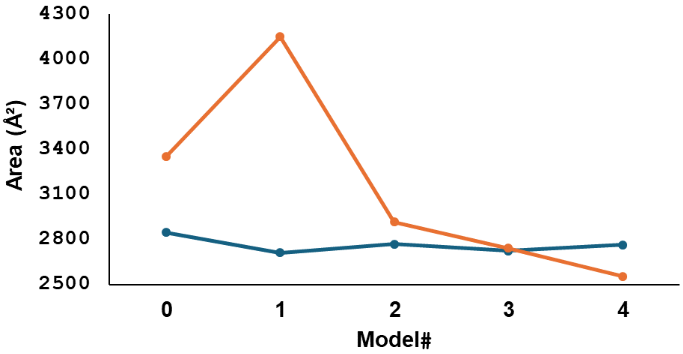

Supplementary Figure S3. Solvent-accessible surface area (SASA) differences between DIO3 (residues 110–304) and SNX6 in complex models. The plots represent AlphaFold3-generated models with different input orders: DIO3 as the first input and SNX6 as the second (orange line), and SNX6 as the first input and DIO3 as the second (blue line).

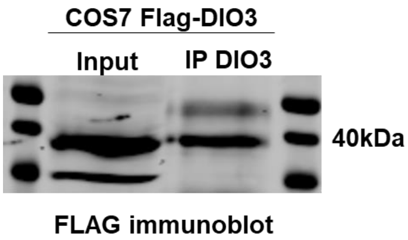

Supplementary Figure S4. WB showing IP of DIO3 in COS7 cells overexpressing C-terminally Flag-tagged mouse DIO3. Anti-Flag antibody was used to visualize DIO3 monomer.

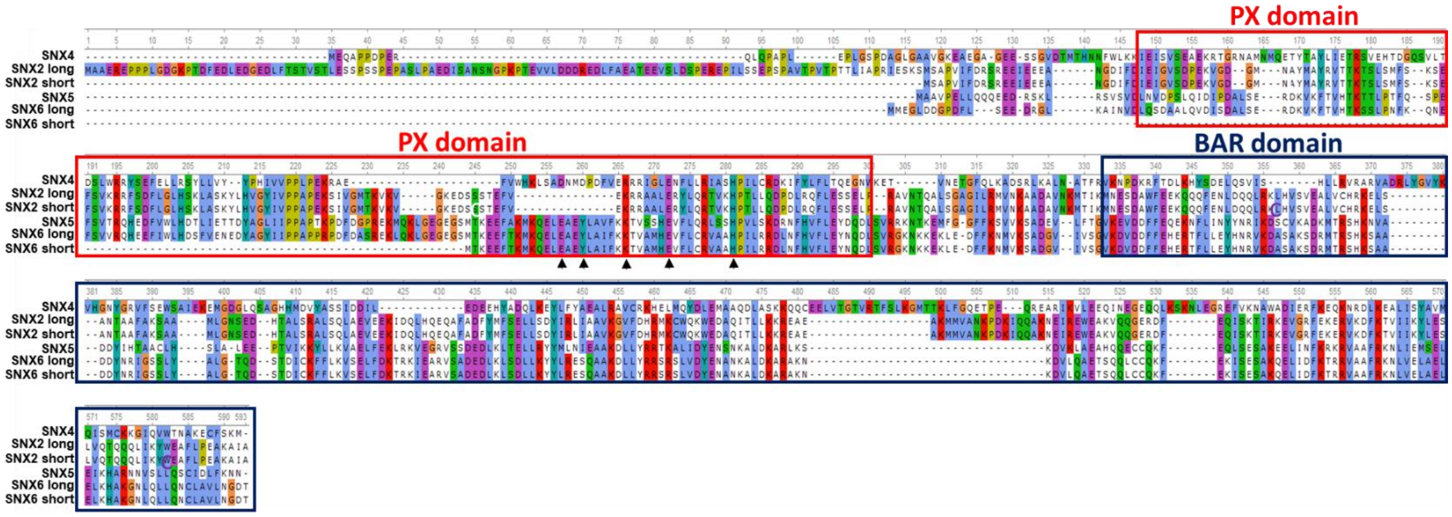

Supplementary Figure S5. Alignments between the various SNXs. PX and BAR domains are highlighted.

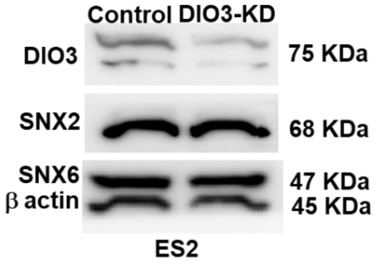

Supplementary Figure S6. Western blot analysis of DIO3, SNX2 and SNX6 in ES2 cells before and after DIO3 silencing. β actin was used as loading control.

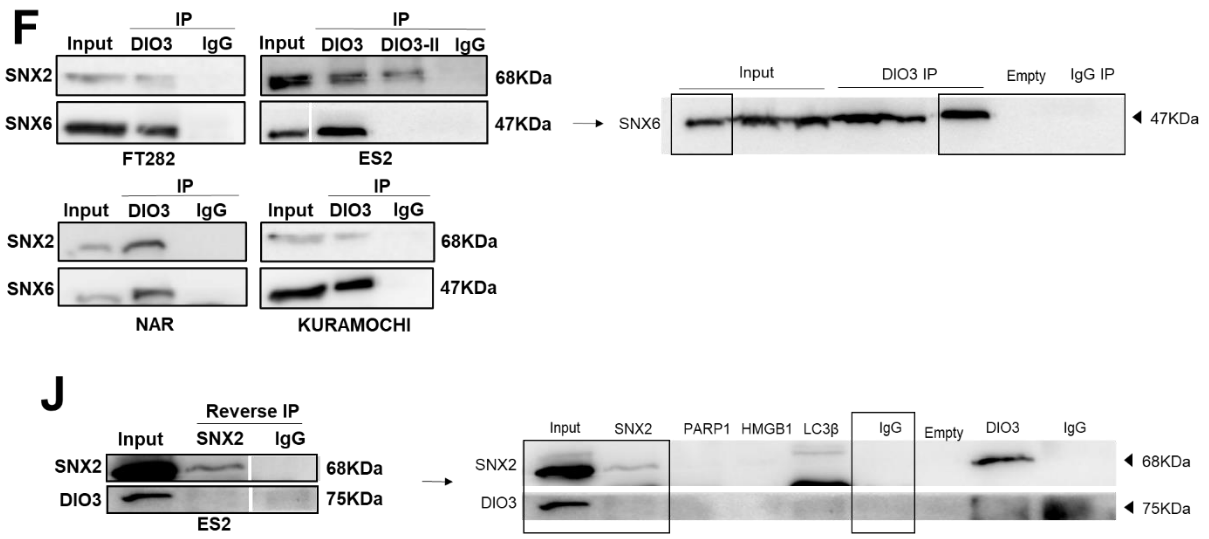

Supplementary Figure S7. Uncropped Western Blots for Fig. 1F and 1J

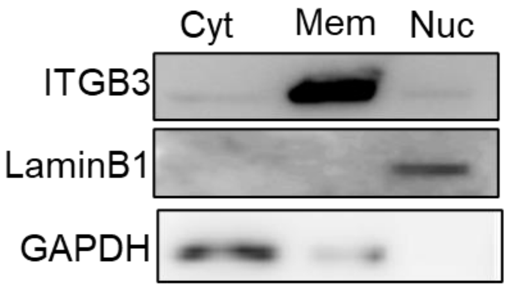

Supplementary Figure S8. Purity of the cytosol (Cyt), membrane (Mem) and the nuclear (Nuc) fractions.

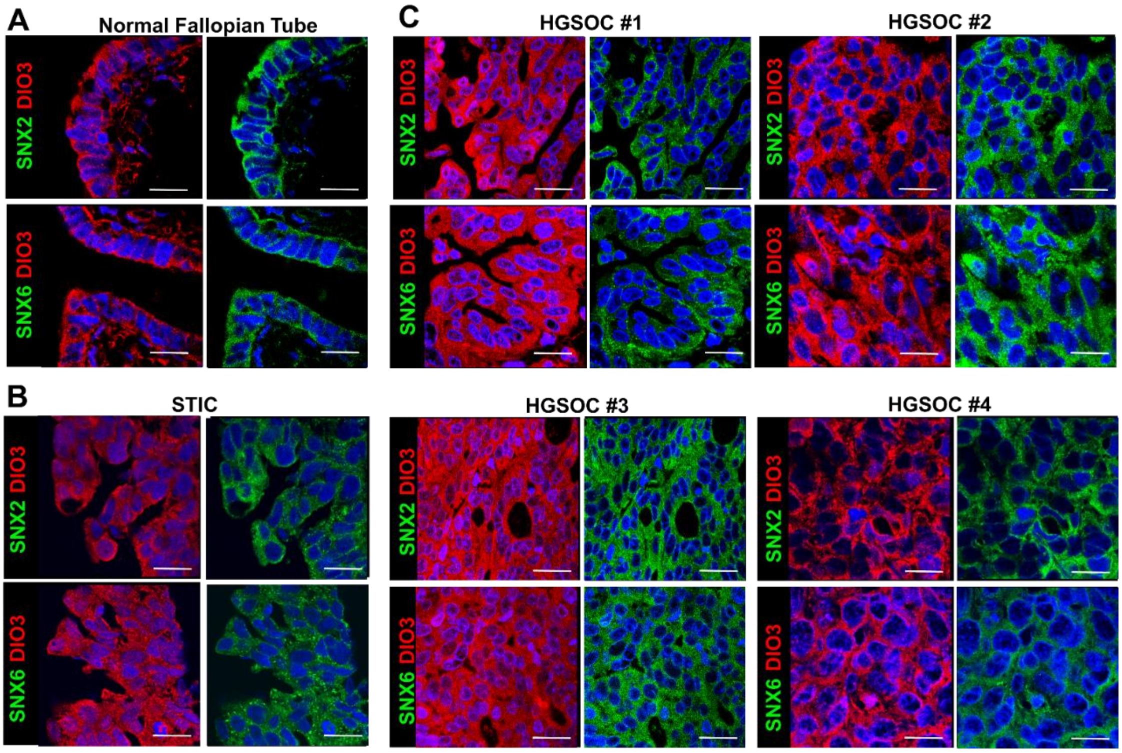

Supplementary Figure S9. DIO3 co-localizes with SNX2 and SNX6 in ovarian cancer cells and human tissues. Separate channels of FPPE tissues from **(A)** normal FT **(B)** premalignant STIC lesions and **(C)** four HGSOC tumors from human patients were stained for SNX2/SNX6 (NL493) and DIO3 (NL557). The nucleus was stained blue with Hoechst 33342. In all images, a single 0.5µm optical section using 63× objective are presented using LeicaSP5 confocal microscopy (scale bar 20µm is presented).

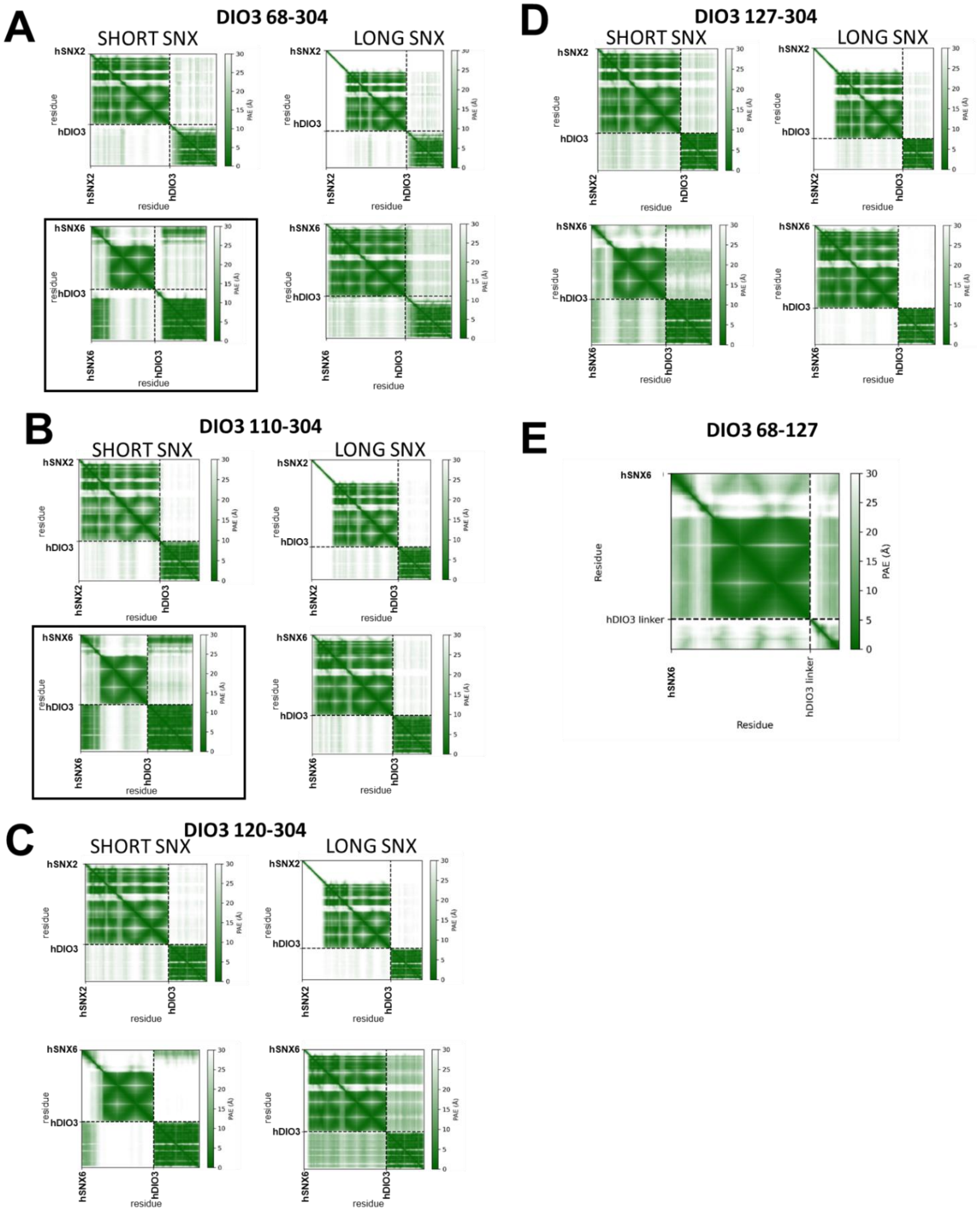

Supplementary Figure S10. Computational modeling of DIO3 interaction with SNX6 and SNX2. Predicted aligned error (PAE) heatmap of the interface between hDIO3 **(A)** 68-304AA **(B)** 110-304AA **(C)** 120-304AA **(D)** 127-304AA and **(E)** 68-127AA with the short or long isoforms of the human SNX2 (upper panels) or SNX6 (lower panels), predicted using AlphaFold3**.**

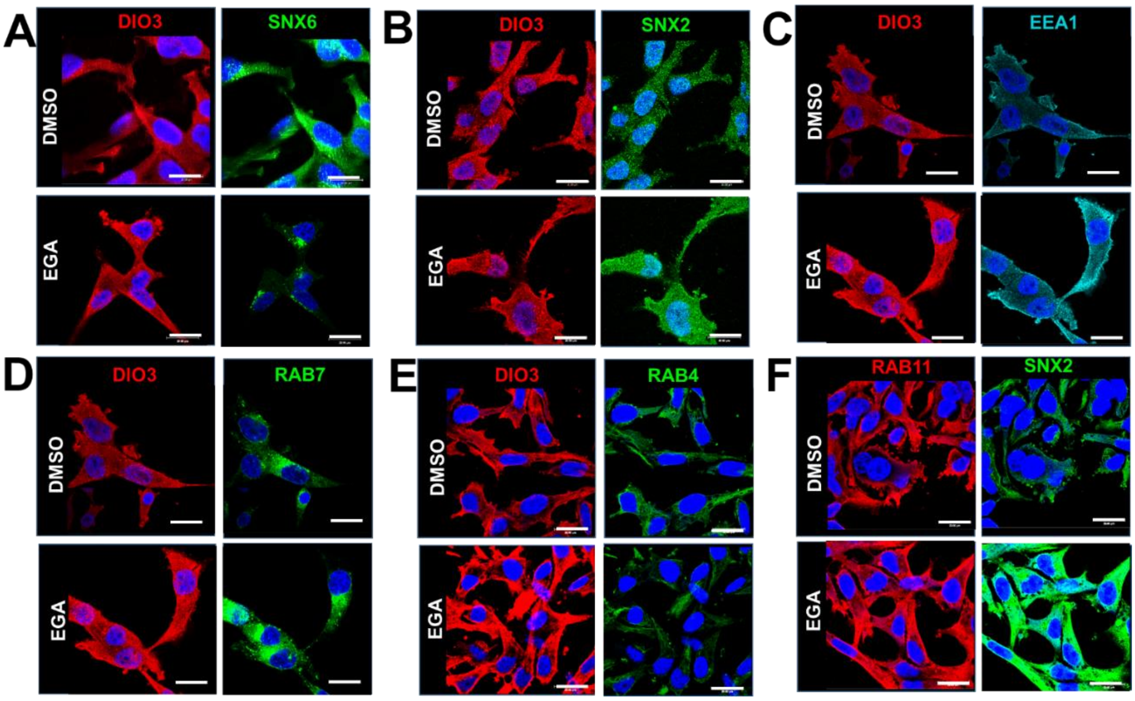

Supplementary Figure S11. SNX2 and SNX6 mediate DIO3 trafficking through a controlled endosome network. Representative separate channels of IF confocal microscopy images of ES2 ovarian cancer cells treated with 50µM EGA for 3 hours showing DIO3 (red, NL557) with **(A)** SNX6 (green, NL493) **(B)** SNX2 (green, NL493) **(C)** Early endosome marker EEA1 (far red, NL637) **(D)** Late endosome marker, RAB7 (green, NL493) **(E)** and RAB4 (green, NL493), a marker of rapid recycling endosome **(F)** SNX2 (green, NL493) and RAB11 (red, NL557), a marker of recycling endosome. In all images Hoechst 33342 (blue) marks the nucleus. Imaging was performed using a Leica SP8 confocal microscope with a 63× objective. Scale bar of 20µm for IF and 15µm for PLA.

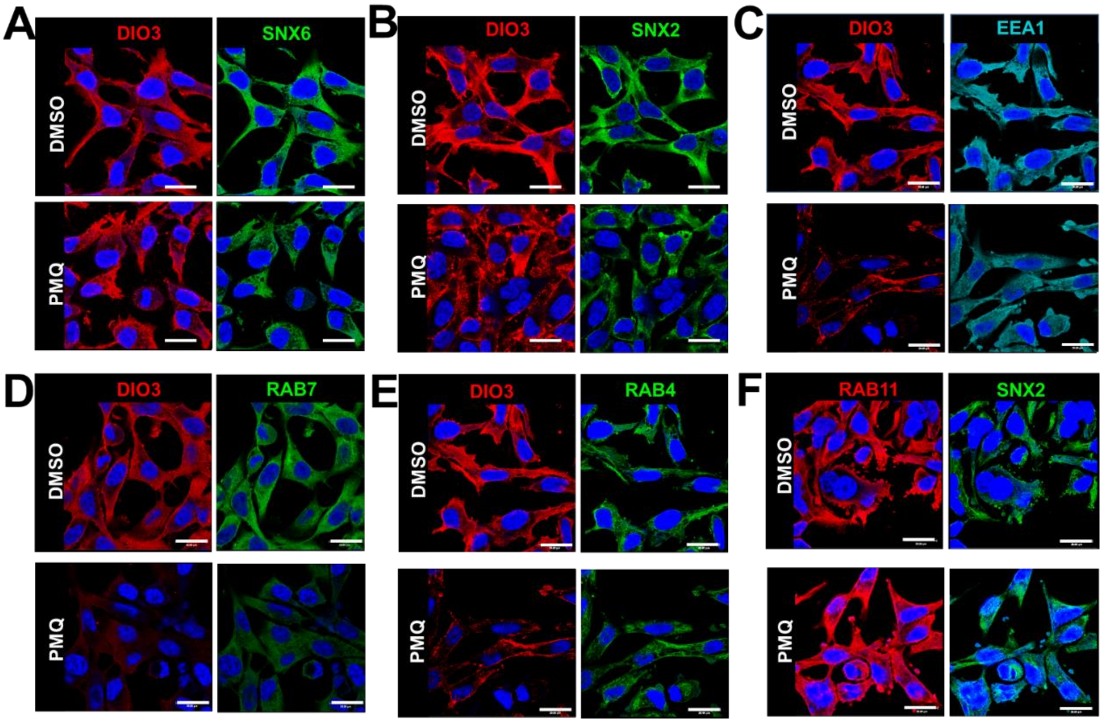

Supplementary Figure S12. DIO3 recycling to the membrane is SNX2 and SNX6-mediated and affects endosomal dynamics Representative separate channels of IF confocal microscopy images of ES2 ovarian cancer cells treated with PMQ for 3 hours showing DIO3 (red, NL557) with **(A)** SNX6 (green, NL493) **(B)** SNX2 (green, NL493) **(C)** Early endosome marker EEA1 (far red, NL637) **(D)** Late endosome marker, RAB7 (green, NL493) **(E)** and RAB4 (green, NL493), a marker of rapid recycling endosome **(F)** SNX2 (green, NL493) and RAB11 (red, NL557), a marker of recycling endosome. In all images Hoechst 33342 (blue) marks the nucleus. Imaging was performed using a Leica SP8 confocal microscope with a 63× objective. Scale bar of 20µm for IF and 15µm for PLA.

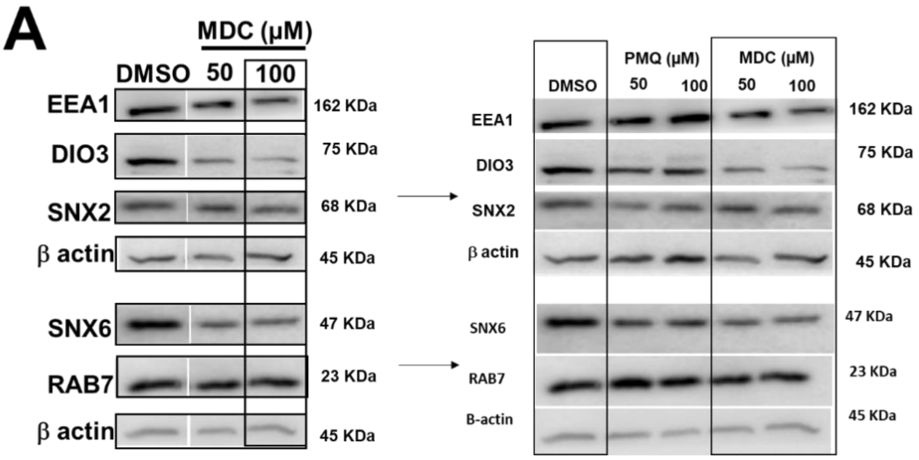

Supplementary Figure S13. Uncropped Western Blots for Fig. 7A.

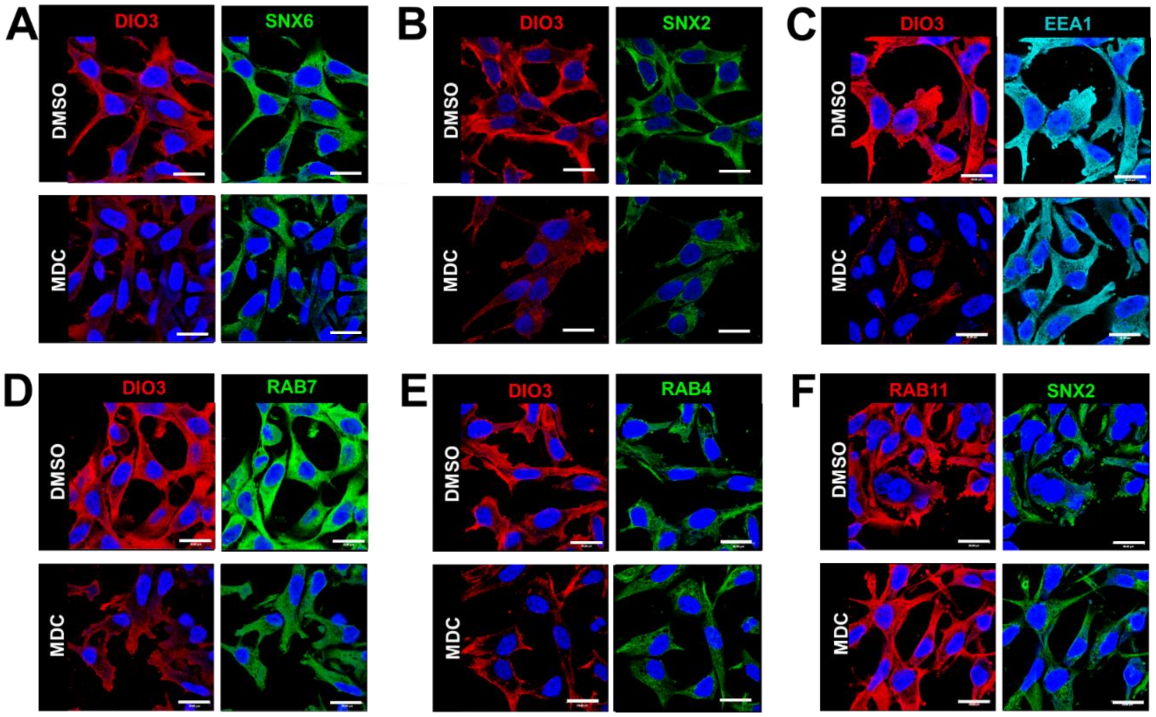

Supplementary Figure S14. DIO3 undergoes endocytosis via SNX6 and SNX2. Representative separate channels of IF confocal microscopy images of ES2 ovarian cancer cells treated with MDC for 3 hours showing DIO3 (red, NL557) with **(A)** SNX6 (green, NL493) **(B)** SNX2 (green, NL493) **(C)** Early endosome marker EEA1 (far red, NL637) **(D)** Late endosome marker, RAB7 (green, NL493) **(E)** and RAB4 (green, NL493), a marker of rapid recycling endosome **(F)** SNX2 (green, NL493) and RAB11 (red, NL557), a marker of recycling endosome. In all images Hoechst 33342 (blue) marks the nucleus. Imaging was performed using a Leica SP8 confocal microscope with a 63× objective. Scale bar of 20μm for IF and 15μm for PLA.
